# Changes over Time in IgE Sensitization to Allergens of the Fish Parasite *Anisakis* spp.

**DOI:** 10.1371/journal.pntd.0004864

**Published:** 2016-07-22

**Authors:** Noelia Carballeda-Sangiao, Ana I. Rodríguez-Mahillo, Mercedes Careche, Alfonso Navas, Ignacio Moneo, Miguel González-Muñoz

**Affiliations:** 1 Department of Immunology, University Hospital La Paz Institute for Health Research, (IdiPaz), Madrid, Spain; 2 Department of Products, Institute of Food Science, Technology and Nutrition, Consejo Superior de Investigaciones Científicas, Madrid, Spain; 3 Department of Biodiversity and Evolutionary Biology, Museo Nacional de Ciencias Naturales, Consejo Superior de Investigaciones Científicas, Madrid, Spain; Albert Einstein College of Medicine, UNITED STATES

## Abstract

**Background:**

Sensitization to *Anisakis* spp. can produce allergic reactions after eating raw or undercooked parasitized fish. Specific IgE is detected long after the onset of symptoms, but the changes in specific IgE levels over a long follow-up period are unknown; furthermore, the influence of *Anisakis* spp. allergen exposure through consumption of fishery products is also unknown.

**Objective:**

To analyse the changes in IgE sensitization to *Anisakis* spp. allergens over several years of follow-up and the influence of the consumption of fishery products in IgE sensitization.

**Methods:**

Total IgE, *Anisakis* spp.-specific IgE, anti-Ani s 1 and anti-Ani s 4 IgE were repeatedly measured over a median follow-up duration of 49 months in 17 sensitized patients.

**Results:**

*Anisakis* spp.-specific IgE was detected in 16/17 patients throughout the follow-up period. The comparison between baseline and last visit measurements showed significant decreases in both total IgE and specific IgE. The specific IgE values had an exponential or polynomial decay trend in 13/17 patients. In 4/17 patients, an increase in specific IgE level with the introduction of fish to the diet was observed. Three patients reported symptoms after eating aquaculture or previously frozen fish, and in two of those patients, symptom presentation was coincident with an increase in specific IgE level.

**Conclusions:**

IgE sensitization to *Anisakis* spp. allergens lasts for many years since specific IgE was detectable in some patients after more than 8 years from the allergic episode. Specific IgE monitoring showed that specific IgE titres increase in some allergic patients and that allergen contamination of fishery products can account for the observed increase in *Anisakis* spp.-specific IgE level.

**Clinical Relevance:**

Following sensitization to *Anisakis* spp. allergens, the absence of additional exposure to those allergens does not result in the loss of IgE sensitization. Exposure to *Anisakis* spp. allergens in fishery products can increase the specific IgE level in some sensitized patients.

## Author Summary

The nematode *Anisakis* spp. is a parasite of marine mammals that can parasitize humans when a raw or undercooked fish containing live *Anisakis* spp. larvae is consumed. As a result, gastrointestinal and/or allergic symptoms are reported. The allergic reaction can be diagnosed by quantifying the serum specific IgE against some parasite allergens. To avoid the appearance of symptoms, sensitized patients are advised to consume frozen or heat-treated fishery products because these treatments kill larvae to prevent new parasitism and allergic reactions. We have analysed the changes in *Anisakis* spp.-specific IgE levels through repeated measures during a long follow-up period of sensitized individuals whose diets did not include fishery products and subjects who regularly consumed fishery products. We have found that IgE sensitization to *Anisakis* spp. allergens persists over several years (more than 8 years in some cases) even in subjects who did not include fishery products into their diets. On the other hand, specific IgE titres increase in some allergic patients during the follow-up period and this increase was coincident with the introduction of previously frozen fish into their diet. These findings indicate that *Anisakis* spp. allergens induce long-lived IgE responses.

## Introduction

The nematode *Anisakis* spp. is a parasite of marine mammals that can parasitize humans when a raw or undercooked fish containing live *Anisakis* spp. L3 is consumed. Ingestion of L3 causes an acute and self-limiting infection that can manifest with abdominal pain, nausea, vomiting or diarrhoea. Infection causes a strong polyclonal humoral immune response, and IgM, IgA, and IgG antibodies are detected after one month of infection [1]. In some patients, an IgE-mediated immune response is also triggered, and in those patients, allergic symptoms, such as urticaria, angioedema and anaphylaxis can develop after sensitization and re-exposure to the allergens of this parasite. The rise in specific IgE is usually accompanied with an increase in total IgE in the first month after the presentation of allergic symptoms, and serial serological analysis of both specific and total IgE values have been proven useful in the diagnosis of gastro-allergic anisakiasis [2]. To avoid the appearance of symptoms, sensitized patients are advised to consume frozen or heat-treated fishery products because these treatments kill larvae to prevent new parasitism [3–5].

Several groups have investigated the kinetics of specific antibody production in experimental animal models [review in 6], but the results of those studies may not be applicable to the human immune responses to this parasite. Studies of the changes over time of the level of specific IgE to *Anisakis* spp. in sensitized patients have shown the persistence of IgE sensitization up to 38 months after the onset of symptoms [1, 2, 4, 7]. However, those studies did not report variations in the specific IgE levels at different follow-up time points.

The aim of this study was to analyse the changes in *Anisakis* spp.-specific IgE levels through repeated measures during a longer follow-up period than previously reported and to compare IgE sensitization between patients whose diets did not include fishery products and subjects who regularly consumed fishery products.

## Methods

### Patients

To analyse the kinetics of the IgE response, *Anisakis* spp.-allergic patients with at least 30 months of follow-up after symptom presentation were selected for this study. A total of 17 patients (six males) with a median age of 53 years (IQR = 45–57 years) were diagnosed as being allergic to *Anisakis* spp. because they reported allergic (urticaria, angioedema or anaphylaxis) and/or gastrointestinal (vomiting, diarrhoea, or abdominal pain) symptoms within 24 h after eating raw or undercooked fish or seafood. One patient reported symptoms after eating cooked fish (scorpion fish cake). Five patients had grass pollen allergy and one of them had dog dander allergy. The data collected at the first visit are shown in Table 1. Allergy was confirmed by a positive prick test and/or detection of specific IgE to *Anisakis* spp. and undetectable levels of IgE to shrimp, *Ascaris lumbricoides*, fish and mites. To assess new sensitization to these allergens, specific IgE levels were quantified at the last visit, and they remained undetectable in all patients. Measurements of the levels of total and specific IgE to *Anisakis* spp. and clinical evaluations were performed during successive visits. All patients were advised at the first visit to avoid consumption of raw and undercooked fish and to eat farmed fish and deep-frozen fishery products; however, patients with levels of specific IgE to *Anisakis* spp. higher than 100 kU/L were initially instructed to consume a fish-free diet for six months.

**Table 1:** Patients’ data at first visit and total and specific IgE values at first and last visits of follow-up.

**Patient**	**Sex**	**Age**	**Symptoms**	**Te (months)**	**Previous allergic episodes**	**Fish**	**Detection of rAni s 1/rAni s 4**	**Follow-up (months)**	**First/last total IgE (kU/L)**	**First/last specific IgE (kU/L)**
1	M	55	U, GI	0.6	n	anchovy	+/+	96	658/605	>100/23.7
2*	M	48	AN, GI	12	n	anchovy, anglerfish	+/-	71	28/20	5/2.1
3	M	63	AG, U, GI	0.6	n	anchovy	+/-	118	1740/437	32.4/21.7
4*	F	30	U, GI	4	n	anchovy	+/-	43	341/41	43.9/3.18
5	M	62	GI	0.7	y	anchovy	+/-	49	541/70	>100/15.7
6	F	60	GI	1	n	hake	+/-	95	1196/384	>100/52.5
7*	F	50	U, GI	1	y	anchovy	+/+	50	1750/71	>100/8.95
8	F	54	U	1	n	anchovy	+/-	61	786/338	>100/37.8
9	F	64	AG, U, GI	5	y	anchovy	+/+	38	213/70	72.6/13.3
10	F	53	AN, U	1	n	anchovy	+/-	44	2958/258	>100/12.1
11*	F	50	U	12	n	anchovy	+/+	38	57/45	4.3/1.1
12	M	27	U	1	n	anchovy	+/+	37	1557/440	82.8/20.1
13	F	54	U, GI	4	y	anchovy	+/-	58	327/103	>100/17.4
14	F	43	U	12	n	scorpion fish cake	+/+	112	557/347	16.1/0.6
15	F	32	U	0.6	n	anchovy	+/-	31	271/478	9.5/6.1
16*	F	53	U, GI	3	n	anchovy	+/-	45	91/11	24.9/2.9
17	M	51	GI	9	n	anchovy	+/-	35	2423/184	79.2/20.6

U: urticaria; GI: gastrointestinal; AN: anaphylaxis; AG: angioedema; Te: Time elapsed from the allergic episode to the first visit; M: male; F: female; n: no; y: yes. Asterisks indicate patients who did not include fish or seafood in their diet during the follow-up period

This study was approved by the Ethics Committee of the Hospital Carlos III (Madrid, Spain), and all included subjects were asked to sign an informed consent form.

### Total and specific IgE

The serum total and specific IgE measurements were performed with a Phadia 250 instrument (Thermo Fischer Scientific, Phadia, Madrid, Spain) according to the manufacturer’s instructions. The detection range for total IgE was 2–5000 kU/L. Regarding positivity for specific IgE antibodies, values >0.7 kU/L [8] were considered positive for IgE to *Anisakis* spp., and values >0.35 kU/L were considered positive for IgE to the other allergens.

### *Anisakis* spp. antigens

Live *Anisakis* spp. larvae in the third stage of the life cycle (L3) were obtained from parasitized hake (*Merluccius merluccius*) at local markets in Madrid, Spain. L3 were extracted from fish tissue, washed in PBS and immediately frozen at -20°C until use. Then, L3 were ground in a Potter-ELV homogenizer and sonicated at 18 w for 5 s. Protein extracts were obtained after centrifugation at 16,000 g and 4°C for 10 min.

Recombinant (r)Ani s 4 and rAni s 1 were obtained as previously reported [9, 10].

### IgE immunoblotting

In addition to the total and specific IgE measurements, IgE immunoblotting was performed with the parasite crude extract, recombinant (r) Ani s 1 and rAni s 4.

Proteins extracted from L3 (15 μg), rAni s 1 (3 μg) and r Ani s 4 (3 μg) were subjected to electrophoresis at 120 V on a 4%-20% Tris-glycine gel (Bio-Rad, Hercules, CA, USA). Thereafter, proteins were transferred to nitrocellulose membranes by applying a constant current of 1.3 A for 7 min in a Trans-Blot Turbo Instrument (Bio-Rad). Membranes were blocked with PBS, 0.05% Tween 20 and 1% BSA for 1 h at room temperature and then incubated with 10 mL of the sera of *Anisakis* spp. -allergic patients (1/20) overnight. After washing with PBS, the membranes were incubated for 2 h with 10 mL of a 1:1000 dilution of a monoclonal anti-IgE antiserum (1 mg/mL; Ingenasa, Madrid, Spain). After additional washes, the membranes were incubated with 10 mL of a 1:20,000 dilution of an alkaline phosphatase–labelled goat anti-mouse antiserum (Sigma-Aldrich, St. Louis, MO, USA). Finally, the membranes were washed and incubated with the BCIP-NBT (Sigma-Aldrich) substrate for 30 min.

### Statistics

Statistical analyses were performed using SPSS 20.0 software (IBM Corporation, NY, USA). Quantitative variables are described as medians and interquartile ranges (IQR). The Mann-Whitney U test was used to compare the values of total IgE and specific IgE quantified at the first and last visits. A paired-samples comparison between the baseline and last visit values was performed using the Wilcoxon signed-rank test. Regression was used to analyse the trends in the changes in specific IgE values. Changes in specific IgE values over time were estimated with linear and non-linear regression models, and the best fit was selected. A p–value of <0.05 was considered statistically significant. For the statistical analysis, all values of *Anisakis* spp.-specific IgE >100 kU/L were assigned a value of 101 kU/L [11].

## Results

At first visit, three patients reported gastrointestinal, allergic (n = 6) or allergic and gastrointestinal symptoms (n = 8) after eating raw or undercooked fish, except P14 who reported urticaria after eating cooked fish (scorpion fish cake). Raw anchovies in vinegar sauce were the most frequently consumed fish related to the onset of symptoms (Table 1). The median time elapsed from the allergic episode to the first visit was one month (IQR = 0.85–7 months). The median follow-up duration was 49 months (IQR = 38–83 months). P5, P7, P9 and P13 reported previous allergic episodes associated with raw fish consumption. At the first visit, using IgE immunoblotting, in addition to *Anisakis* spp.-specific IgE, specific IgE to rAni s 1 was detected in all patients, and specific IgE to rAni s 4 was detected in six patients (Table 1). Although patients were advised to eat aquaculture and previously frozen fish, five patients did not include fish or seafood in their diet during the follow-up period (P2, P4, P7, P11 and P16), and they were considered to have not been re-exposed to *Anisakis* spp. antigens or allergens.

Positive specific IgE to *Anisakis* spp. values (>0.7 kU/L) were detected in all patients throughout the follow-up period, except for P14, who had a specific IgE to *Anisakis* spp. value of 0.6 kU/L at the end of a 112-month follow-up period (Table 1). Baseline total IgE (557 kU/L, IQR = 242–1648 kU/L) and *Anisakis* spp.-specific IgE values (79 kU/L, IQR = 20–101 kU/L) were significantly higher than the final total IgE value (184 kU/L, IQR = 57–410 kU/L; p <0.01) and the *Anisakis* spp.-specific IgE titre (13 kU/L, IQR = 3–21 kU/L, p< 0.01) (Table 1). The median value of the specific IgE decrease was 76% over the follow-up period (IQR = 60%-88%). The paired-samples comparison between the measurements at baseline and the last visit showed significant decreases in both total IgE (p < 0.01) and *Anisakis* spp.-specific IgE titres (p < 0.01). No significant differences were found between the patients who were and were not re-exposed to *Anisakis* spp. in the decrease in the *Anisakis* spp.-specific IgE level over the follow-up period (76%, IQR = 51%-84% and 88%, IQR = 66%-92%, respectively; p = 0.28) or in the follow-up duration (53 months, IQR = 37–96 and 45 months, IQR = 40–60, respectively; p = 0.72).

**Fig 1:**
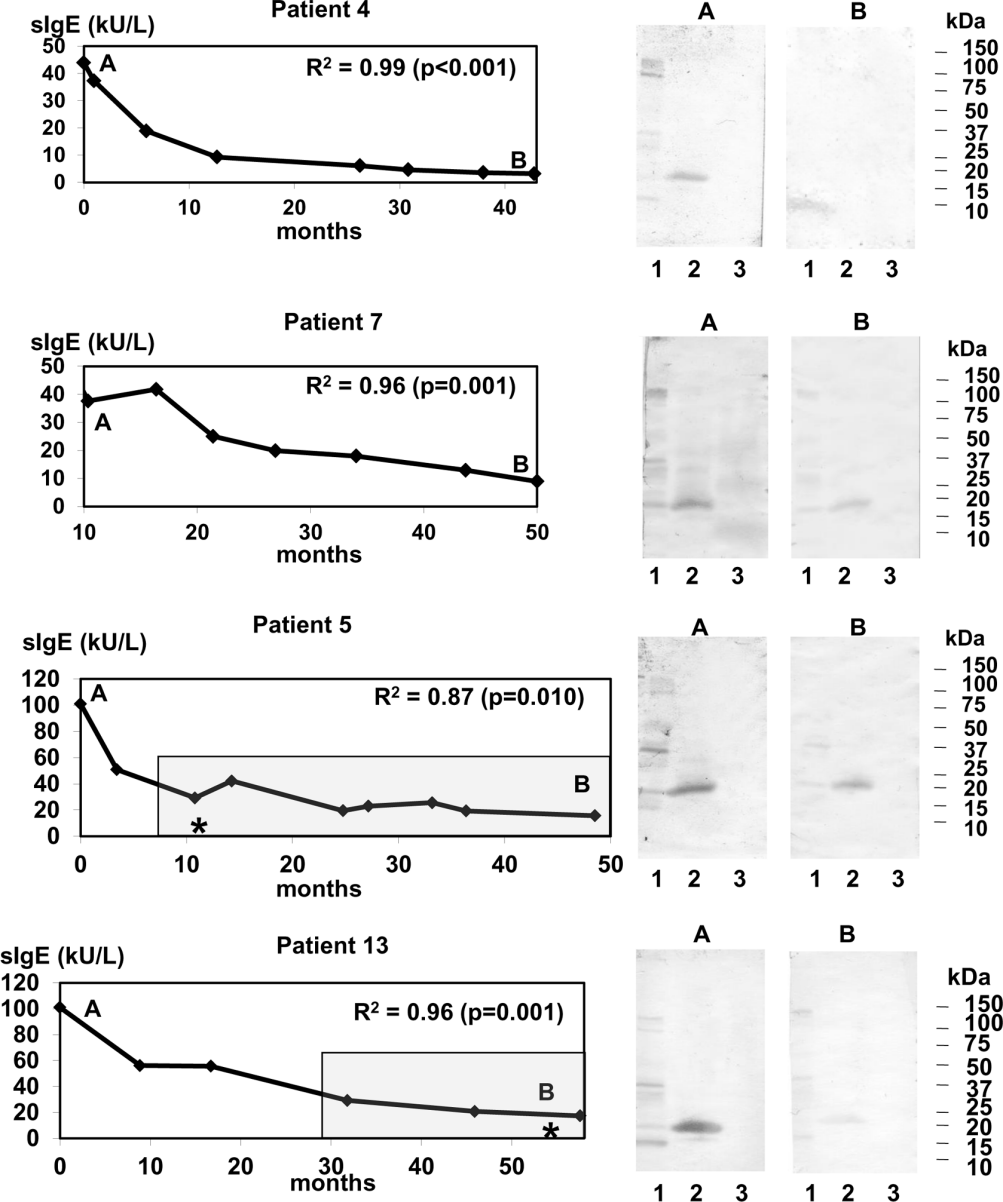
Changes during follow-up in the *Anisakis* spp.-specific IgE level fit a non-linear regression. Persistence of *Anisakis* spp.-specific IgE (sIgE) in patients who were not re-exposed (P4 and P7) and those who were re-exposed (P5 and P13) to *Anisakis* spp. allergens are depicted. The adjusted coefficient of determination (R^2^) and the p values are shown. Letters indicate the time points during follow-up when IgE immunoblotting was performed. Lane 1: *Anisakis* spp. crude extract; lane 2: rAni s 1; lane 3: rAni s 4. The shadow box indicates the follow-up period during which the patients were consuming fish. Asterisks mark the time points at which symptoms associated with the intake of fish were reported. sIgE: specific IgE to *Anisakis* spp.

**Fig 2:**
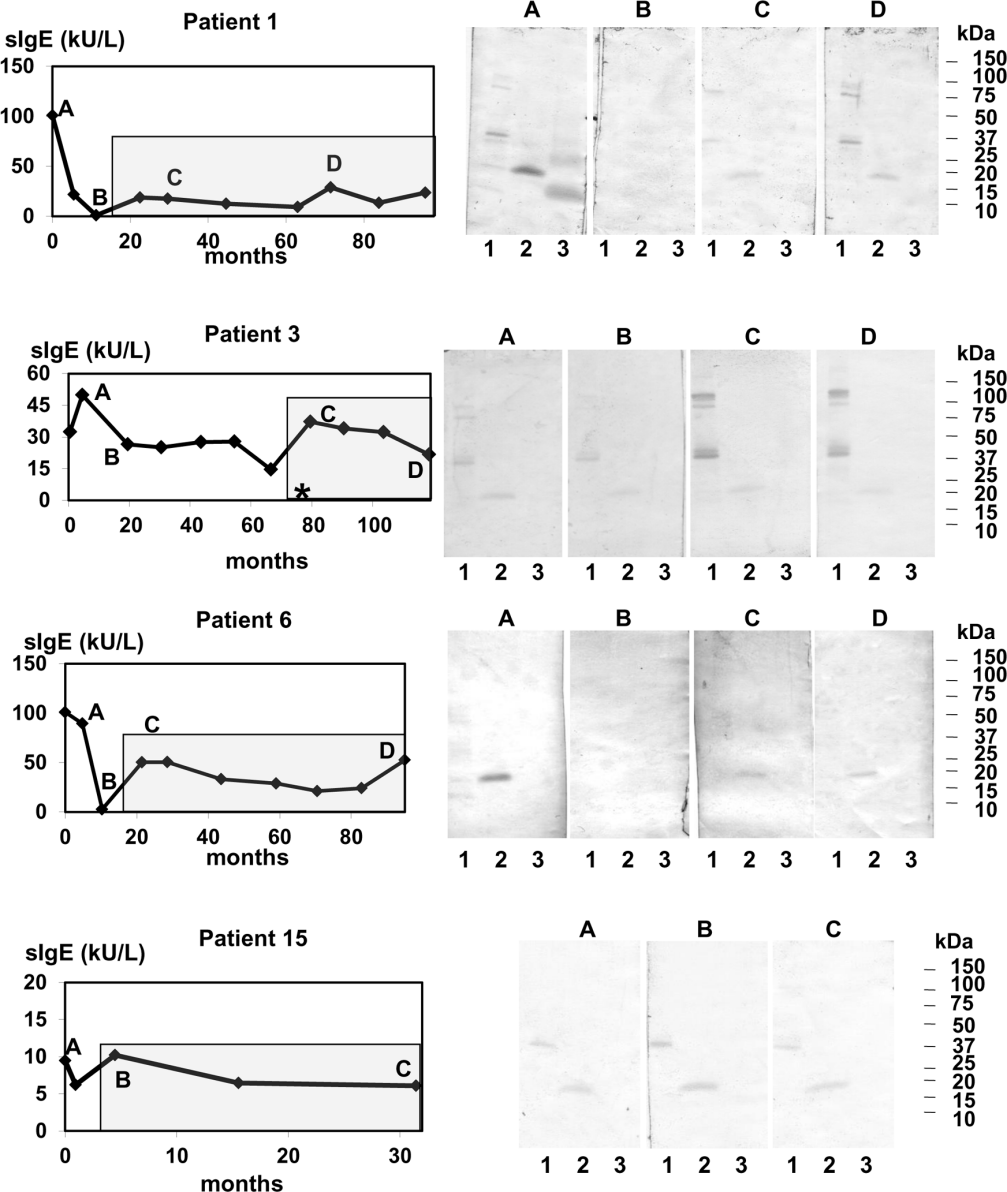
Changes in IgE sensitization in the patients who experienced increases in *Anisakis* spp.-specific IgE (sIgE) level during follow-up. Letters indicate the time points of follow-up when IgE immunoblotting was performed. Lane 1: *Anisakis* spp. crude extract; lane 2: rAni s 1; lane 3: rAni s 4. The shadow box indicates the follow-up period during which the patients were consuming fish. The asterisk marks the time point during which symptoms associated with the intake of fish was reported. sIgE: specific IgE to *Anisakis* spp.

Regression was used to analyse the trend in the changes in *Anisakis* spp.-specific IgE values. The *Anisakis* spp.-specific IgE values underwent an exponential or polynomial decay trend in 13/17 patients, including the patients who had not included fish in their diet during the follow-up period (Fig 1 and S1 Fig). However, the changes in the *Anisakis* spp.-specific IgE levels from baseline to last visit in four patients (P1, P3, P6 and P15) did not fit a regression model (Fig 2). The *Anisakis* spp.-specific IgE level in P1 decreased to 1 kU/L at 10 months, and then increased and remained at > 12 kU/L throughout the remainder of the follow-up period. The *Anisakis* spp.-specific IgE level in P3 initially showed a 70% decrease (from 50 kU/L to 14 kU/L) and, then, increased up to 37 kU/L at one year later. The changes in the *Anisakis* spp.-specific IgE level in P6 were similar to those in P1, with a decrease of specific IgE titre until reaching 3 kU/L at 12 months and an obvious increase at the next visit (50 kU/L). P15 showed a moderate level of *Anisakis* spp.-specific IgE (10 kU/L) at the first visit; it decreased at one month (6 kU/L) and increased at the following visit. The *Anisakis* spp.-specific IgE increases in these four patients were coincident with the introduction of aquaculture and frozen fish into their diet (Fig 2). The paired samples comparisons in these patients revealed no significant differences between baseline total IgE (927 kU/L, IQR = 367–1604 kU/L) and last visit total IgE (457 kU/L, IQR = 397–573 kU/L; p = 0.27) or between baseline *Anisakis* spp.-specific IgE (66 kU/L, IQR = 15->100 kU/L) and last visit *Anisakis* spp.-specific IgE (23 kU/L, IQR = 10–45 kU/L; p = 0.07). When the total and specific IgE titres and the duration of follow-up for the patients whose data fit a regression model were compared to those of these four patients, only total IgE at last visit was found to be higher in the latter group (184 kU/L, IQR = 57–410 kU/L vs. 457 kU/L, IQR = 397–573 kU/L, p < 0.01).

Three patients reported symptoms during the follow-up period. P3 experienced localized acute urticaria in the hands after eating cod that had been frozen for 72 h at home (Fig 2). P5 reported diarrhoea after eating aquaculture sea bass (Fig 1). In these two patients, the appearance of symptoms was coincident with an increase in *Anisakis* spp.-specific IgE level. P3 and P5 did not report new episodes in the subsequent visits. P13 showed generalized acute urticaria after eating commercial frozen hake (Fig 1). Unfortunately, this episode occurred at the end of the follow-up period, and we could not assess if the level of IgE against *Anisakis* spp. had varied. Other common causes of acute urticaria were discarded.

To determine if the changes in specific IgE over time are related to changes in the recognition pattern of allergens, IgE immunoblotting was performed at different time points. As expected, baseline IgE immunoblotting showed different patterns for the parasite crude extracts [12]. rAni s 1 was detected by all patients, and rAni s 4 was detected by six patients (Figs 1 and 2 and S1 Fig). The intensities of the bands corresponding to rAni s 1 paralleled the *Anisakis* spp.-specific IgE levels. During the follow-up period, the rAni s 4 bands disappeared prior to the rAni s 1 bands, suggesting that Ani s 4 is an early marker of *Anisakis* spp. infection. On the other hand, the increase in *Anisakis* spp.-specific IgE over time observed in some patients was associated with an increase in the intensity of some proteins in the parasite crude extract and rAni s 1 (Fig 2).

## Discussion

Our study analysed the changes in the values of total IgE and specific IgE to *Anisakis* spp. over time using a longer follow-up period (31–118 months) than previous studies (6–38 months) [1, 2, 4, 7]. Repeated measures were performed throughout the study period and included detection of Ani s 1 and Ani s 4. Ani s 1 is a major and heat stable allergen [12] and Ani s 4 is a pepsin and heat-resistant allergen, and its clinical relevance has been verified because it is associated with anaphylaxis [9]. Exposure to the live fish-borne parasite *Anisakis* spp. L3 can produce an acute and self-limiting infection in humans with allergic and gastrointestinal symptoms. To avoid the appearance of symptoms, patients are advised to consume frozen or heat-treated fishery products because these treatments kill larvae and thus prevent new parasitism [3–5]. An alternative is to consume aquaculture fish because the risk of exposure to *Anisakis* spp. larvae in farmed fish has been shown to be minimal, because no *Anisakis* spp. larvae have been found in their viscera or flesh [5, 13]. Twelve patients regularly consumed aquaculture and frozen fish during the study period, and five patients decided to stop consuming fish throughout the follow-up period. The decrease in the *Anisakis* spp.-specific IgE levels in these patients, who were considered to have not been re-exposed to parasite material, was higher than those found previously in patients on a fish-free diet (57%) with a follow-up period of 13 months, and this discrepancy was probably due to our longer follow-up period [4]. On the other hand, we did not observe significant differences in the total or *Anisakis* spp.-specific IgE values between the patients who did and did not consume fish during the follow-up period [4]; thus, the rate of *Anisakis* spp.-specific IgE decay does not seem to be influenced by the consumption of previously frozen fish.

Our results show that IgE sensitization to *Anisakis* spp. allergens persists over several years *since specific IgE was detectable in some patients after more than 8 years from the allergic episode*. Similar results were observed in a nine-year follow–up study of adult subjects sensitized to food allergens [14]. In our study, specific IgE to Ani s 1 was detectable up to 118 months from the onset of symptoms, and this result agrees with that obtained in a study with 6–38 months of follow-up [7] and suggests that Ani s 1 can be detected in both recent and old *Anisakis* spp. infection cases. Therefore, the persistent IgE sensitization observed in *Anisakis* spp. allergic patients who were not exposed to parasite allergens for several years indicates that *Anisakis* spp. allergens induce long-lived IgE responses.

The analysis of the changes over time in specific IgE level in the patients that were not re-exposed to *Anisakis* spp. allergens and in some patients who regularly consume aquaculture and frozen fish shows that the decrease in specific IgE titres fit a non-linear regression model. However, we observed that the *Anisakis* spp.-specific IgE level in four patients (P1, P3, P6 and P15) increased at some time points during follow–up. This increase in *Anisakis* spp.-specific IgE could be due to sensitization to other allergen sources that have been reported to cross-react with *Anisakis* spp. allergens, i.e., other parasite nematodes, mites and crustaceans [15–17]. However, according to the available patient data, this explanation does not seem to be valid because our patients were not sensitized to allergens that cross-react with *Anisakis* spp. allergens at baseline, and no new onset hypersensitivity to those allergens was found during the follow-up period. In addition, the increase in *Anisakis* spp.-specific IgE was parallel to the increase in Ani s 1 detected by immunoblotting, which supports the hypothesis that the observed changes in *Anisakis* spp.-specific IgE were actually due to exposure to *Anisakis* spp. allergens. Another explanation for this observation is re-infection with live L3. However, we do not believe this is the case because the patients reported symptoms after eating frozen or aquaculture fish, and it is unlikely that an acute parasitism episode went unnoticed by these patients, as they had experienced one previously. On the other hand, previously unrecognized allergens have been detected in the course of gastro-allergic anisakiasis [1], which was not observed in our patients according to the results of IgE immunoblotting. Accordingly, it has been shown that adult patients allergic to aeroallergens did not acquire sensitization to new allergens, but they exhibited a pre-established profile of allergens after antigen exposure. The levels of allergen-specific IgE in previously sensitized allergic patients decreased in the absence of allergen exposure and increased upon allergen exposure [18]. Because the raise in specific IgE levels is coincident with the introduction of fishery products to the diet, a more plausible explanation for the changes in IgE sensitisation is that exposure to allergens present in those fishery products is involved in the increase in the *Anisakis* spp.-specific IgE level. Some *Anisakis* spp. antigens have been shown to be stable and to maintain their capacity to bind IgE after different freezing and heat treatments [19, 20]. Furthermore, *Anisakis* spp. antigens have been detected in farmed salmon and processed fish products [21], which could contribute to the variations in the trends of IgE sensitization observed in this study.

The clinical significance of these results is difficult to determine because very few patients (n = 3) reported symptoms after the initial acute parasitism episode in our study. It has been proposed that the high rate of *Anisakis* spp. parasitism of fish in our region would result in frequent contact with parasite material, which would cause symptoms in sensitized subjects exposed to it when previously frozen or heat-treated fish is included in their diet [22]. However, our results suggest that the exposure to *Anisakis* spp. antigens present in fishery products may contribute to the persistence and even to the increase in *Anisakis* spp.-specific IgE level. The increase in the *Anisakis* spp.-specific IgE level associated with the appearance of symptoms indicates that exposure to *Anisakis* spp. material could be involved in the onset of symptoms in some *Anisakis* spp. allergic patients [12]. However, more studies are required to evaluate the clinical relevance of the re-exposure to *Anisakis* spp. in sensitized patients.

In conclusion, we have shown that IgE sensitization to *Anisakis* spp. allergens can last more than 8 years. Specific IgE monitoring showed that specific IgE titres increase in some allergic patients and that allergen contamination of fishery products can account for the observed increase in *Anisakis* spp.-specific IgE level.

## Supporting Information

S1 Checklist STROBE checklist. (DOCX)

S1 Fig Changes in IgE sensitization to *Anisakis* spp. allergens during follow-up. Letters indicate the time points during follow-up when IgE immunoblotting was performed. Lane 1: *Anisakis* spp. crude extract; lane 2: rAni s 1; lane 3: rAni s 4. The shadow box indicates the follow-up period during which the patients were consuming fish. sIgE: specific IgE to *Anisakis* spp.
(PDF)

S1 Text Accession numbers. (DOC)
