# Supplementary material for: Changes over Time in IgE Sensitization to Allergens of the Fish Parasite Anisakis spp
Source: PLoS Negl Trop Dis. 2016 Jul 22;10(7):e0004864. doi: 10.1371/journal.pntd.0004864 (PMC4957799; doi:10.1371/journal.pntd.0004864)
Supplement: S1 Fig — Letters indicate the time points during follow-up when IgE immunoblotting was performed. Lane 1: Anisakis spp. crude extract; lane 2: rAni s 1; lane 3: rAni s 4. The shadow box indicates the follow-up period during which the patients were consuming fish. sIgE: specific IgE to Anisakis spp. (PDF) [file pntd.0004864.s002.pdf]

Figure S1

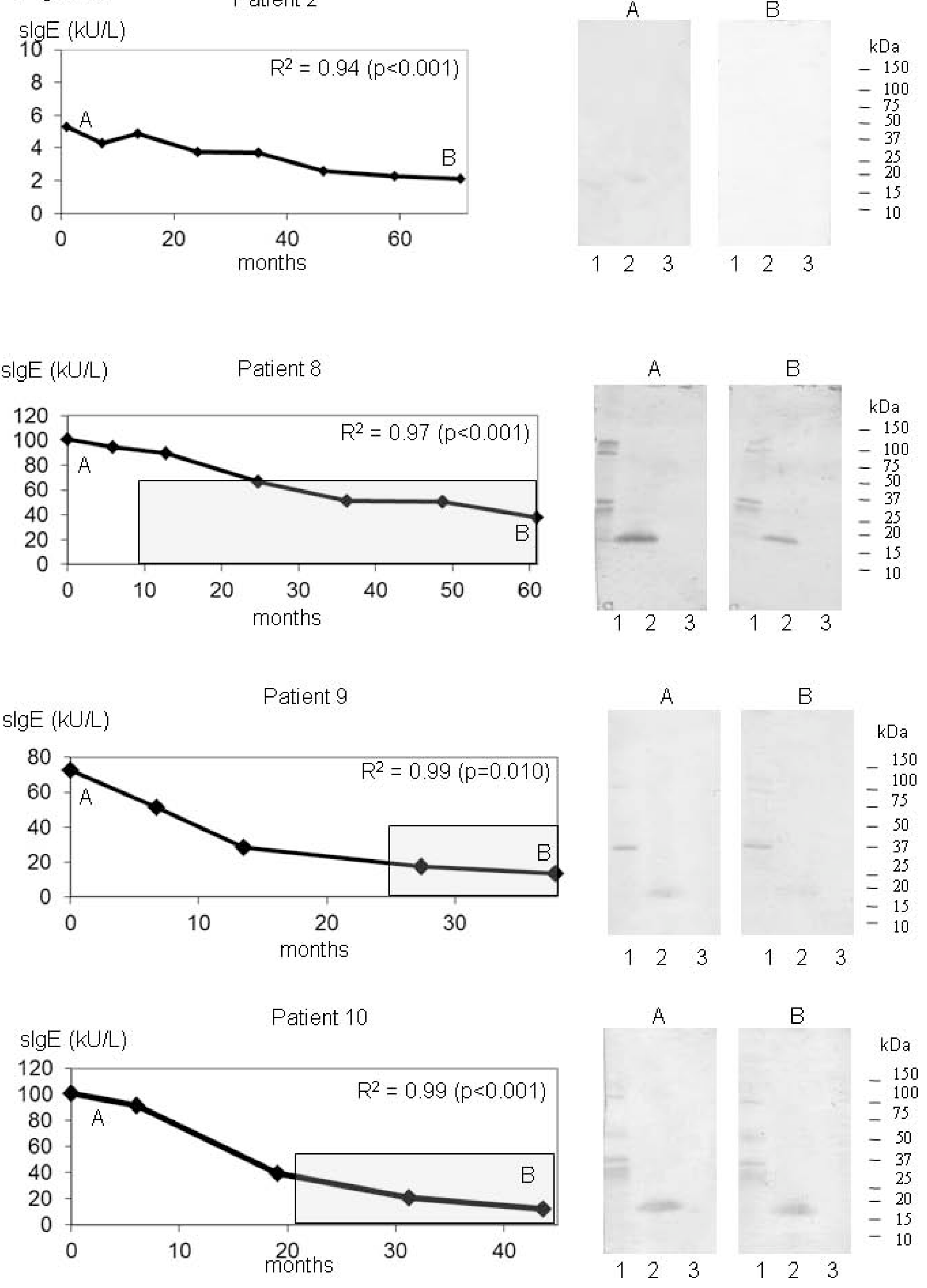

Patient 2
sIgE (kU/L)
R² = 0.94 (p<0.001)
A
B
months
kDa
150
100
75
50
37
25
20
15
10
1 2 3
Patient 8
sIgE (kU/L)
R² = 0.97 (p<0.001)
months
Patient 9
sIgE (kU/L)
R² = 0.99 (p=0.010)
months
Patient 10
sIgE (kU/L)
R² = 0.99 (p<0.001)
months

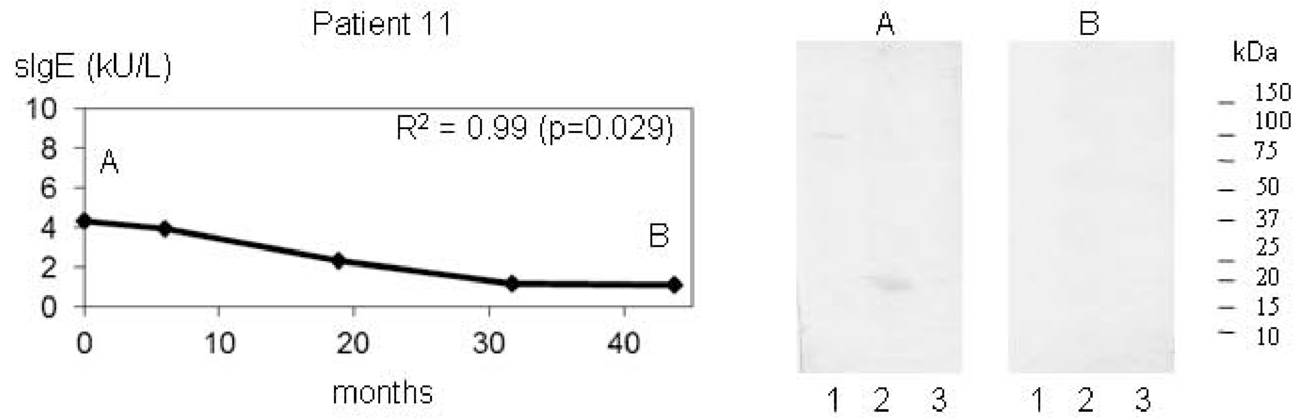
Patient 11
sIgE (kU/L)
10
8
6
4
2
0
R² = 0.99 (p=0.029)
A
B
0
10
20
30
40
months
A
B
kDa
150
100
75
50
37
25
20
15
10
1 2 3
1 2 3

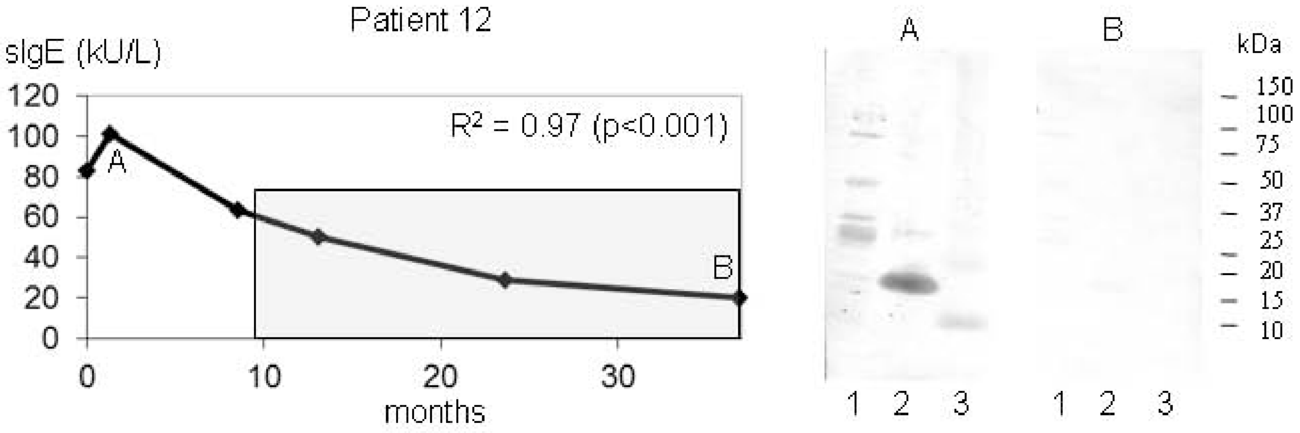
Patient 12
sIgE (kU/L)
120
100
80
60
40
20
0
R² = 0.97 (p<0.001)
A
B
0
10
20
30
months
A
B
kDa
150
100
75
50
37
25
20
15
10
1 2 3
1 2 3

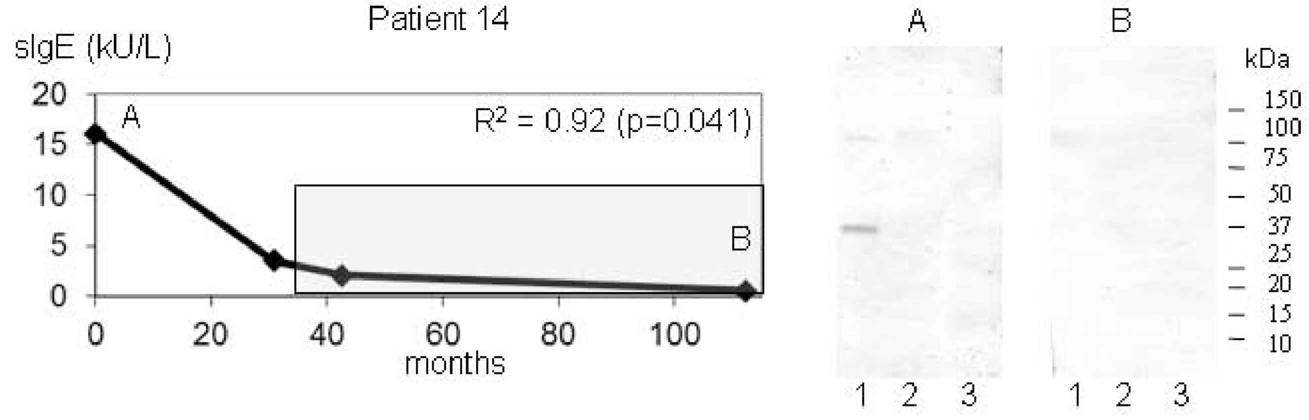
Patient 14
sIgE (kU/L)
20
15
10
5
0
R² = 0.92 (p=0.041)
A
B
0
20
40
60
80
100
months
A
B
kDa
150
100
75
50
37
25
20
15
10
1 2 3
1 2 3

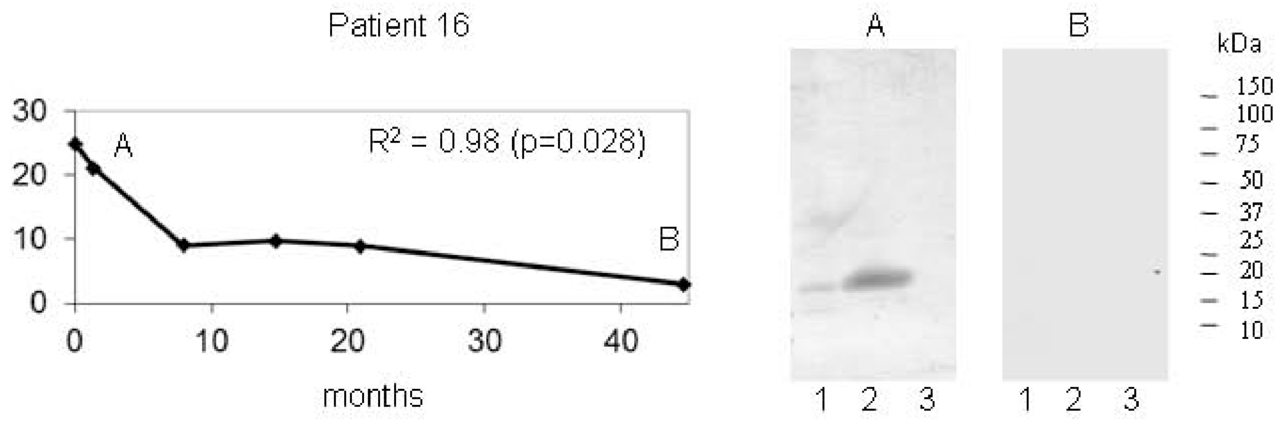
Patient 16
30
20
10
0
R² = 0.98 (p=0.028)
A
B
0
10
20
30
40
months
A
B
kDa
150
100
75
50
37
25
20
15
10
1 2 3
1 2 3

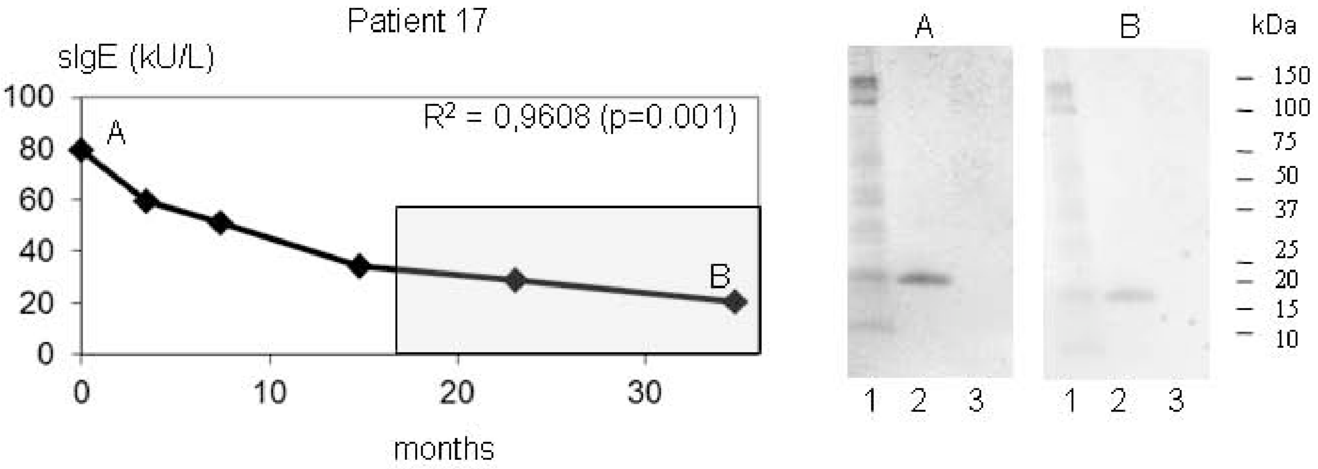

Patient 17
sIgE (kU/L)
100
80
60
40
20
0
R² = 0,9608 (p=0.001)
A
B
0
10
20
30
months
A
B
kDa
150
100
75
50
37
25
20
15
10
1 2 3
1 2 3
