# Supplementary material for: Changes over Time in IgE Sensitization to Allergens of the Fish Parasite Anisakis spp
Source: PLoS Negl Trop Dis. 2016 Jul 22;10(7):e0004864. doi: 10.1371/journal.pntd.0004864 (PMC4957799; doi:10.1371/journal.pntd.0004864)
Supplement: S1 Text — (DOC) [file pntd.0004864.s003.doc]

List of accession numbers

Ani s 1: accession no. Q7Z1K3

Ani s 4: accession no. Q14QT4
